# Supplementary figures and images for: Genome-wide analysis of transcription factors related to anthocyanin biosynthesis in carmine radish (Raphanus sativus L.) fleshy roots
Source: PeerJ. 2019 Nov 7;7:e8041. doi: 10.7717/peerj.8041 (PMC6842556; doi:10.7717/peerj.8041)

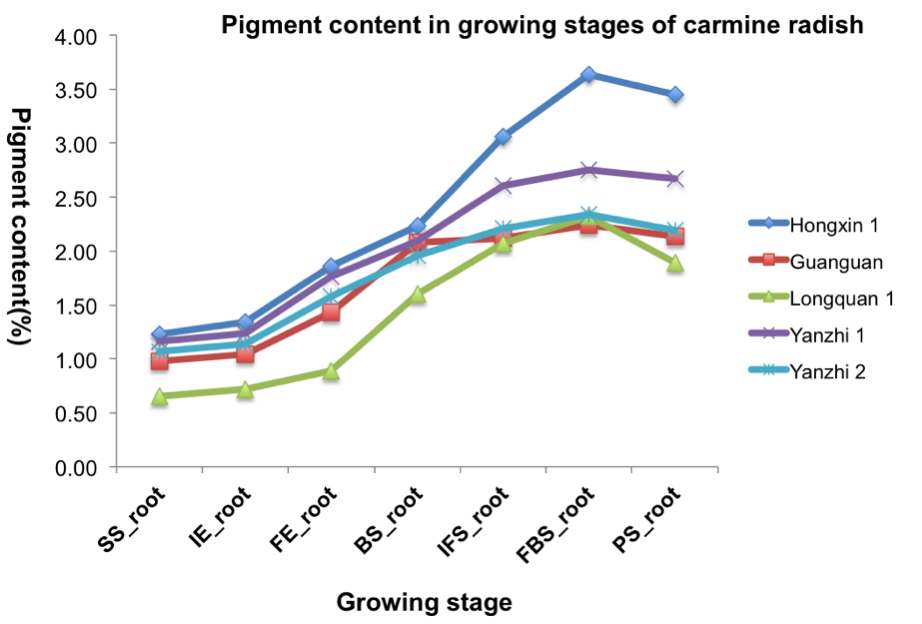

Supplement: Supplemental Information 1 [file peerj-07-8041-s001.png]
